# *Vibrio cholerae* O1 El Tor and O139 Bengal Strains Carrying *ctxB*^ET^, Bangladesh

**DOI:** 10.3201/eid1910.130626

**Published:** 2013-10

**Authors:** Shah M. Rashed, Anwarul Iqbal, Shahnewaj B. Mannan, Tarequl Islam, Mahamud-ur Rashid, Fatema-tuz Johura, Haruo Watanabe, Nur A. Hasan, Anwar Huq, O. Colin Stine, R. Bradley Sack, Rita R. Colwell, Munirul Alam

**Affiliations:** International Center for Diarrhoeal Disease Research, Bangladesh (ICDDR,B), Dhaka, Bangladesh (S.M. Rashed, A. Iqbal, S.B. Manna, T. Islam, M.-u. Rashid, F.-t. Johura, M. Alam);; National Institute of Infectious Diseases, Tokyo, Japan (H. Watanabe); University of Maryland College Park, Maryland, USA (N.A. Hasan, A. Huq, R.R. Colwell);; University of Maryland Baltimore, Maryland, USA (C. Stine);; Johns Hopkins Bloomberg School of Public Health, Maryland, USA (R.B. Sack, R.R. Colwell).

**Keywords:** Vibrio cholerae, ctxB^ET^, ctxB^CL^, altered El Tor, prototype El Tor, antibiotic resistance, PFGE, pulsed-field gel electrophoresis, bacteria

**To the Editor.** Cholera, caused by *Vibrio cholerae*, continues to affect millions of persons in disease-endemic areas where safe drinking water is scarce and sanitation is poor. Of 7 cholera pandemics recorded since 1817, *V. cholerae* serogroup O1 classical (CL) biotype was associated with the sixth, whereas the seventh (ongoing) pandemic was initiated by *V. cholerae* O1 biotype El Tor (ET), which displaced CL in the early 1960s (*1*). During 1992–1993, a *V. cholerae* non-O1 serogroup, designated *V. cholerae* O139 synonym Bengal, initiated cholera epidemics in India and Bangladesh by transiently displacing *V. cholerae* O1 ET biotype (*2*). *V. cholerae* O139 was less frequently associated with cholera in Bangladesh than *V. cholerae* ET in 1994 and the years following, until 2005 (*3*); it has been undetected since then. Meanwhile, *V. cholerae* ET has shown genetic changes since 2001, and isolates carry the *ctxB* gene of the CL biotype (*ctxB*^CL^) in Bangladesh (*4*). Although the genetic transition from *ctxB*^ET^ to *ctxB*^CL^ was observed during 1998–1999 for *V. cholerae* O139 (*5*), *V. cholerae* strains carrying *ctxB*^ET^ were considered extinct, i.e., undetected for about a decade.

During June 2010–December 2012, the International Centre for Diarrheal Disease Research, Bangladesh (ICDDR,B) systematically conducted ongoing epidemiologic ecologic surveillance in Dhaka, Chhatak, and Mathbaria and isolated *V. cholerae* strains (n = 500 [clinical/environmental]: Dhaka [n = 110/94], Mathbaria [n = 90/79], Chhatak [n = 111/16]). Of the 500 *V. cholerae* isolates, 496 were confirmed as O1 and 4 as O139 Bengal, on the basis of serologic, phenotypic, and genetic properties (*3*,*6*–*8*). All *V. cholerae* O1 and O139 isolates were positive for *ctxA*, *tlc*, *ace*, and* zot* and possessed ET biotype–specific markers *tcpA*^ET^, *hlyA*^ET^, and *rtxC*. Mismatch amplification mutation assay–PCR (*9*) demonstrated *ctxB*^CL^ allele in 492 *V. cholerae* O1 ET strains (altered ET), whereas *ctxB*^ET^ was found in 8 isolates (4 *V. cholerae* O1 ET and 4 *V. cholerae* O139).

Nucleotide sequencing of *ctxB* showed that the translated sequences of *V. cholerae* O1 and O139 strains carrying *ctxB*^ET^ were identical to those of the ET reference strain N16961 (GenBank accession no. NC_002505), with tyrosine and isoleucine at positions 39 and 68, respectively, as opposed to altered ET, which possesses histidine and threonine at positions 39 and 68, respectively (*4*). PCR additionally showed that the *V. cholerae* O1 and O139 Bengal strains carrying *ctxB*^ET^ had the ET biotype–specific RS1 element gene *rstC* and repressor gene *rstR*^ET^, suggesting prototype ET attributes (*7*).

Three *V. cholerae* strains carrying *ctxB*^ET^ were first isolated in 2011 from surface water: one O1 strain and one O139 strain from Mathbaria and one O1 strain from Chhatak. In 2012, *V. cholerae* O1 carrying *ctxB*^ET^ was isolated from cholera patients in Mathbaria and Chhatak (n = 1 each). Also, 3 O139 strains carrying *ctxB*^ET^ were isolated from surface water in Dhaka. The confirmed *V. cholerae* O1 and O139 Bengal strains carrying *ctxB*^ET^ were of particular interest because altered ET strains carrying *ctxB*^CL^ have been deemed the cause of endemic cholera in Bangladesh since 2001 (*4*) and globally (*10*).

*V. cholerae* strains carrying *ctxB*^ET^ were closely related to the pre-2001 *V. cholerae* strains carrying *ctxB*^ET^, as were the O139 Bengal strains carrying *ctxB*^ET^. Two lines of evidence support this close relationship. First, the antimicrobial drug resistance patterns of 3 of the *V. cholerae* O139 strains isolated in Dhaka during 2012 were resistant to trimethoprim/sulfamethoxazole (25 µg), whereas the remaining O139 and 4 O1 strains were susceptible to all drugs tested, including azithromycin (15 µg), ciprofloxacin (5 µg), gentamicin (10 µg), ampicillin (10 µg), tetracycline (30 µg), and erythromycin (15 µg). Second, pulsed-field gel electrophoresis (PFGE) of *Not*I-digested genomic DNA showed identical banding patterns for the 4 *V. cholerae* O1 strains carrying *ctxB*^ET^ and the pre-2001 ET strains, including N16961, and the DNA pattern differed from that of the altered ET associated with endemic cholera in Bangladesh (Figure). All 4 *V. cholerae* O139 strains had typical O139 Bengal banding patterns, shown by PFGE, except that 1 strain had an extra band (Figure). Comparison of PFGE patterns with those of previously isolated *V. cholerae* O139 strains (1993–2005) showed that recently isolated strains (2011–2012) belonged to 1 of the ancient clones, suggesting that the strain has been present in Bangladesh since 1993 (Figure).

**Figure:**
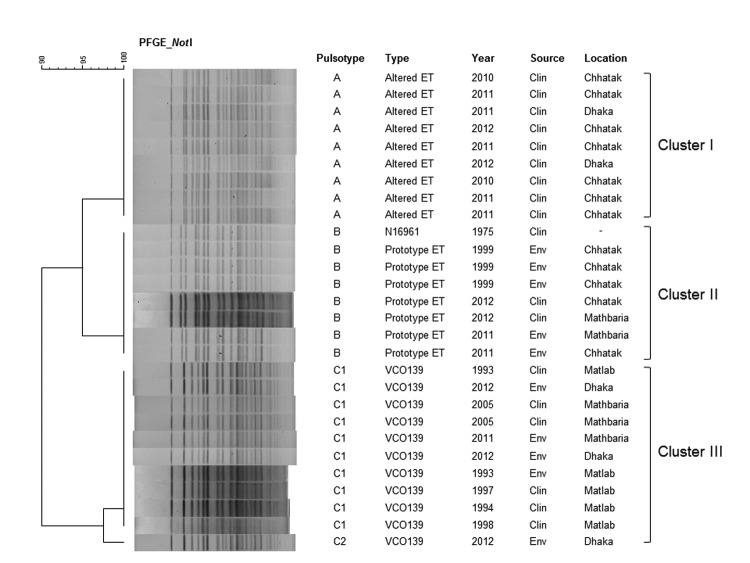
DNA fingerprinting patterns of *Vibrio cholerae*. Dendrogram was prepared by Dice similarity coefficient and UPGMA (unweighted pair-group method with arithmetic mean) clustering methods by using pulsed-field gel electrophoresis (PFGE) images of the *Not*I-digested genomic DNA. The scale bar at the top (left) indicates the correlation coefficient (range 90%–100%). *V. cholerae* altered ET (*ctxB*^CL^) strains (pulsotype A) formed a major cluster (cluster I), separated from prototype ET (*ctxB*^ET^) strains (cluster II; pulsotype B) and *V. cholerae* O139 strains (cluster III; pulsotype C), suggesting that they are genetically different. ET, El Tor; Clin, Clinical; Env, environmental.

In conclusion, we provide evidence of the coexistence of *V. cholerae* O1 and O139 strains, which shows that strains carrying *ctxB*^ET^, not isolated for approximately a decade in Bangladesh, have again been isolated (*3*). Although the epidemiologic importance of the observed genetic change in the *ctxB* is yet to be understood, the finding of *V. cholerae* strains carrying *ctxB*^ET^ in surface water of Bangladesh in 2011 and in association the following year with cholera may be yet another turning point, considering that the global pattern of cholera is changing rapidly.
